# Aqueous humour extracellular vesicle membrane protein profiling reveals pathological features of refractory macular edema

**DOI:** 10.3389/fcell.2026.1880885

**Published:** 2026-09-01

**Authors:** Yufei Wu, Yujie Wang, Xiaodong Xie, Shuanghua Xin, Weina Ren, Yitian Zhao, Zai-Long Chi, Qinkang Lu

**Affiliations:** 1 School of Ophthalmology and Optometry, Wenzhou Medical University, Wenzhou, Zhejiang, China; 2 Ningbo Key Laboratory for Neuroretinopathy, The Affiliated People’s Hospital of Ningbo University, Ningbo, Zhejiang, China

**Keywords:** artificial intelligence, biomarker discovery, diabetic macular edema, extracellular vesicle membrane proteins, macular edema

## Abstract

**Background:**

Both diabetic macular edema (DME) and retinal vein occlusion-related macular edema (RVO-ME) can become refractory to anti-vascular endothelial growth factor (anti-VEGF) therapy, but the underlying mechanisms are unclear. Molecular discrimination of refractory disease could guide personalized treatment. This study examined whether aqueous humor-derived extracellular vesicle (EV) membrane proteins can characterize refractoriness and reveal etiology-specific pathways.

**Methods:**

This prospective cohort study included 28 patients with DME or RVO-ME (14 each), further divided into treatment-naïve and refractory subgroups. Aqueous humour samples were collected before intravitreal anti-VEGF injection. EV membrane proteins were profiled using an EV Array chip targeting 435 antibodies. Differentially expressed proteins were analyzed by bioinformatics, including Gene Ontology, Kyoto encyclopaedia of genes and genomes (KEGG) pathway enrichment, Gene set enrichment analysis (GSEA), and cell-of-origin mapping using public single-cell RNA-seq data.

**Results:**

VEGF/VEGFR2 were elevated in treatment-naïve DME and RVO-ME. Refractory DME showed upregulation of C5 and CD34 (complement/immune activation). Refractory RVO-ME exhibited increased CD68 and Annexin A1 with decreased PDGFR (chronic inflammation, vascular dysregulation). RANTES was commonly upregulated in refractory disease. Several EV proteins discriminated refractory cases with high accuracy (AUC 0.898–0.980). Cellular origin suggested immune cell-derived EVs in DME, retinal cell-derived EVs in RVO-ME. External validation confirmed key differences.

**Conclusion:**

Refractory ME involves distinct pathways: immune-inflammatory activation in DME versus chronic inflammation with vascular dysregulation in RVO-ME. EV membrane proteins from aqueous humor provide insights into therapeutic resistance and hold promise as biomarkers for personalized treatment decisions.

## 1 Introduction

Macular edema (ME) is the primary pathological mechanism underlying vision loss in patients with diabetic retinopathy (DR) (Tan et al., 2017) and retinal vein occlusion (RVO) (Hayreh, 2021). The current global prevalence of diabetic macular edema (DME) is estimated to be 19 million people and is projected to increase to 29 million by 2045 (Teo et al., 2021); with RVO, the number of patients experiencing vision impairment is anticipated to be even greater. Intraretinal fluid accumulation can lead to the disruption of crucial structures such as the external limiting membrane and ellipsoid zone, resulting in irreversible tissue damage (Daruich et al., 2018; Ehlers et al., 2019; Borrelli et al., 2023; do Nascimento et al., 2024) that ultimately leads to a loss of central vision, visual distortion, and reduced contrast sensitivity (Tomkins-Netzer et al., 2015). In some cases of chronic ME, elevated intraocular pressure may ensue, potentially triggering neovascular ocular diseases (Hayreh, 2007; Hayreh et al., 2011; Hayreh, 2021) and even retinal and vitreous haemorrhages. These pathological processes contribute to severe visual impairment and significantly compromise patients’ quality of life. Nonetheless, the link between medical treatment results and molecular changes in ME has not been fully explained to date, resulting in inconsistencies in clinical practice regarding when treatment is initiated and what strategies are used for monitoring and retreatment (Hui et al., 2022).

Currently, intravitreal injection of anti-vascular endothelial growth factor (anti-VEGF) drugs is the first-line treatment for DME and retinal vein occlusion–related macular edema (RVO-ME). However, responses to anti-VEGF agents can vary considerably, with a significant proportion of patients experiencing suboptimal or unsustainable outcomes (Zhang et al., 2022; Chen et al., 2025). In these cases, macular edema recurs within weeks or months after injection, even with a consistent treatment schedule, and treatment-related complications, such as neuroretinal atrophy, may occur (Podkowinski et al., 2019). In addition, there is no standardised definition, diagnostic standard or biomarker for “nonresponse” to treatment, and there is no protocol for switching to second-line or adjuvant treatments (Ng et al., 2025). This clinical phenomenon suggests that the pathophysiology of ME is complex and multifactorial (Torres-Costa et al., 2020); that the VEGF pathway may not be the sole driver of ME; and that more complex, poorly understood molecular mechanisms contribute to resistance to anti-VEGF therapy as ME progresses. Consequently, unravelling the intrinsic processes underlying the transition from treatment-naïve to refractory ME has emerged as a critical scientific question for overcoming current therapeutic limitations.

Intraocular fluid, especially aqueous humour (AH), is increasingly being considered a valuable source of liquid biopsies. It can be used to understand the pathological state of the eye in real time (Wolf et al., 2023; Chigane et al., 2025) and has been employed for accurate diagnosis and detection of ophthalmic diseases (Hillier et al., 2018; Laíns et al., 2019; Hou et al., 2020). Previous studies have partially explored changes in metabolites and proteins in the AH of ME patients compared with healthy controls (Jiang et al., 2023; Jin et al., 2025). However, neither the intrinsic relationship between refractory and treatment-naïve ME nor the proteomic differences across various disease states of macular edema have been systematically investigated (Xiong et al., 2021; Han et al., 2022; Jiang et al., 2023; Zhang et al., 2024). The proteome directly reflects gene functional outcomes, serving as a critical bridge connecting the genome to disease phenotypes and accurately mirroring the pathophysiological status of the body (Wolf et al., 2023; Rejas-González et al., 2024; Knol et al., 2025). Extracellular vesicles (EVs), which carry specific membrane proteins from their source cells, effectively transmit molecular information between cells, offering an unprecedented window for the *in vivo*, noninvasive exploration of disease mechanisms. We hypothesised that the membrane proteomic profile of aqueous humour EVs likely captures key information throughout the progression of ME from onset to treatment resistance and holds promise as a molecular biomarker for the early identification of refractory ME.

Artificial intelligence is advancing rapidly in ophthalmology, particularly in predictive modeling and multi-omics integration. This trend has generated a growing demand for well-annotated molecular features that are clinically accessible and readily integrable into machine learning pipelines. Aqueous humour, as a liquid biopsy medium, provides a readily accessible source of such features. However, current AI applications remain predominantly reliant on imaging data and largely overlook the molecular drivers of treatment resistance. We hypothesized that the membrane proteomic profile of aqueous humour EVs captures critical information throughout the progression of ME from onset to treatment resistance. This profile may also hold promise as a molecular biomarker for the early identification of refractory ME. The resulting dataset is intended not only to clarify divergent pathophysiology but also to provide a prioritized, biologically interpretable feature panel that could support the future development of AI-assisted diagnostic tools.

On this basis, our study focused on DME and RVO-ME, each categorised into “treatment-naïve” and “refractory” stages to represent disease progression, aiming to identify pathological mechanisms associated with specific disease states across different aetiologies and stages. Moreover, we determined whether a common proteomic pattern underpinned the transition of ME to a refractory state. These findings provide a solid basis for the discovery of novel biomarkers and the development of targeted therapeutic strategies.

## 2 Methods

### 2.1 Study design

**FIGURE 1:**
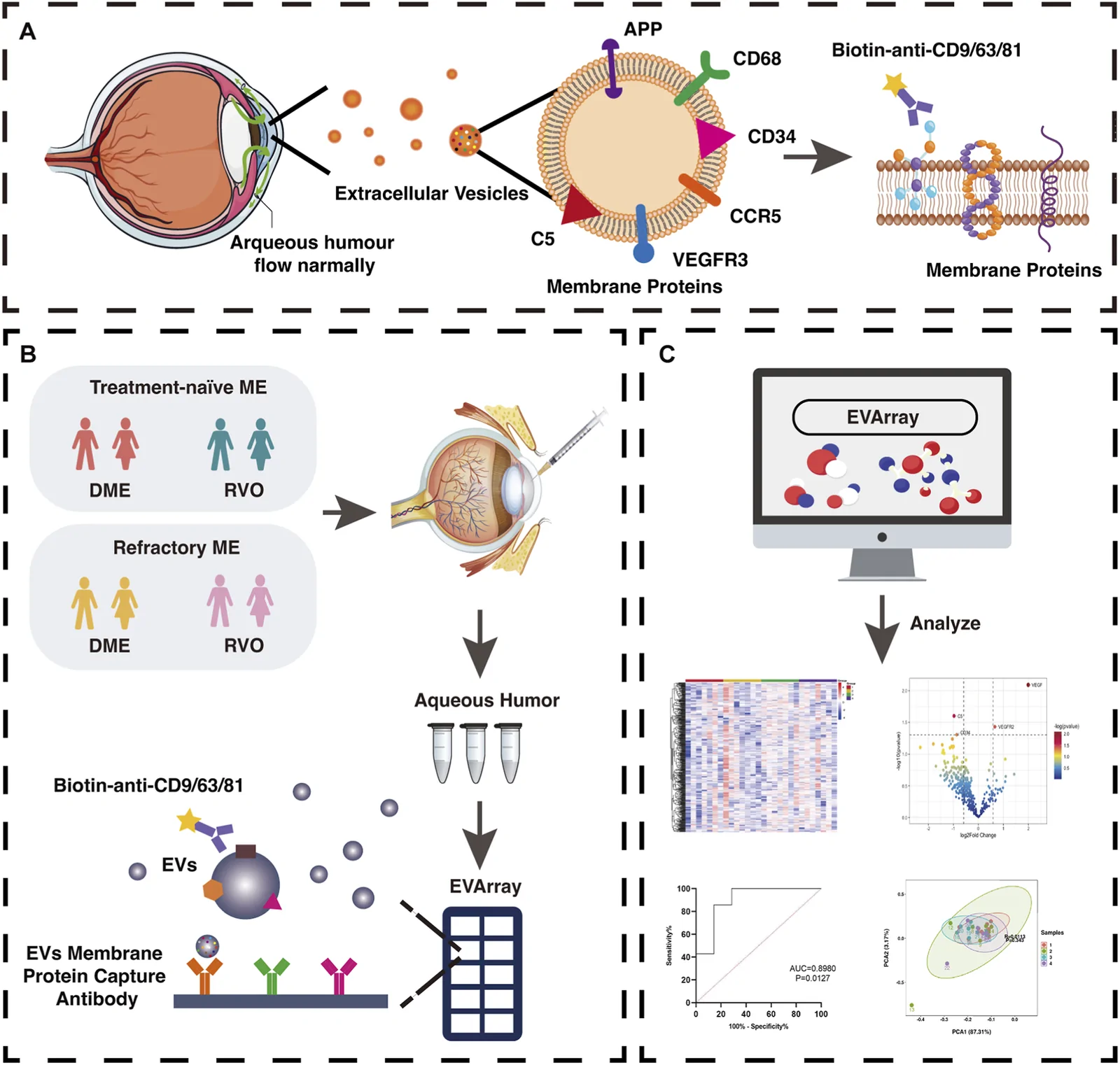
Schematic overview of the study design and the molecular transition from treatment-naïve to refractory ME. **(A)** AH is rich in various bioactive molecules, including EVs. Among these, EVs with diameters of approximately 30–200 nm possess membrane proteins that exhibit multiple bioactive functions. **(B)** Design of the research workflow. AH was collected from patients receiving intravitreal injections of anti-VEGF drugs. EV Array technology, which is based on antibody array technology, uses biotin-labelled antibodies such as CD63, CD9, and CD81 to detect the relative expression levels of EV membrane proteins. **(C)** Bioinformatics-based functional analysis of the aqueous humour–derived EV membrane proteome using raw data obtained via EV Array technology. ME, macular edema; AH, aqueous humour; EV, extracellular vesicle.

This prospective cohort study included 28 patients with macular edema who were enrolled in the Ophthalmology Center at Ningbo University Affiliated People’s Hospital (Ningbo, Zhejiang Province, China) from June 2023 to May 2024. The participants included 14 patients with DME and 14 patients with RVO-ME (Figure 1).

Patients who were receiving their first intravitreal treatment with anti-VEGF drugs were defined as the treatment-naïve group, and those with persistent or recurrent edema after several treatments (at least three intravitreal injections of anti-VEGF agents or steroids) were defined as the refractory group (Shah and Maturi, 2017; Madjedi et al., 2022; Liu et al., 2024); each subgroup contained seven patients. All patients underwent comprehensive ophthalmic examinations before treatment, with aqueous humour samples collected before intravitreal injections. Clinical diagnoses were established by experienced retinal specialists at the Ophthalmology Center of Ningbo University Affiliated People’s Hospital on the basis of angiography and comprehensive ophthalmic examinations. The same technician performed all the specialised ophthalmic measurements.

### 2.2 Inclusion and exclusion criteria

The inclusion criteria for the DME group were as follows: (1) Patients were over 50 years old but not more than 70 years old. (2) Patients had been diagnosed with type 2 diabetes mellitus. (3) Patients had been diagnosed with DME on the basis of imaging evidence (fundus fluorescein angiography, FFA/optical coherence tomography, OCT). (4) Patients fulfilled the definition of “treatment-naïve ME” or “refractory ME” as stated above. The inclusion criteria for the RVO group were as follows: (1) Patients were aged above 50 years but not more than 70 years (2) Patients had been diagnosed with RVO on the basis of imaging evidence (FFA/OCT), and other systemic conditions (such as diabetes or infection) had been excluded. (3) Patients fulfilled the definition of “treatment-naïve ME” or “refractory ME” as stated above.

Patients meeting any of the following criteria were excluded: (1) History of diagnosed ocular complications, including but not limited to primary open-angle glaucoma, primary angle-closure glaucoma, age-related macular degeneration, high myopic choroideraemia, active uveitis, and central serous chorioretinopathy, among others. (2) History of ocular surgery or infection, including open-eye trauma, prior ocular surgery, ocular infection, or ocular inflammation before or during the study period. (3) Ocular surgery, such as cataract or vitreoretinal surgery, within the past 3 months (4) Severe systemic diseases, such as cardiovascular disease, liver cirrhosis, renal failure, malignancy, or a history of organ transplantation. (5) Contraindications for angiography: known allergy to sodium fluorescein or indocyanine green or other contraindications preventing FFA or Indocyanine Green Angiography (ICGA) examinations.

All patients underwent comprehensive ophthalmic and systemic examinations during the initial visit and follow-up, including slit-lamp biomicroscopy, intraocular pressure (IOP) measurement, best-corrected visual acuity measurement, ultrawide-angle fundus photography, OCT, and fluorescein sodium and indocyanine green angiography (FFA and ICGA, respectively). All OCT scans were centred on the fovea, and the device automatically calculated the central macular thickness (CMT) within 1 mm of the fovea.

### 2.3 Collection of aqueous humour and use of EV array

Following enrolment, all patients underwent anterior chamber paracentesis using a 1-mL syringe before receiving an intravitreal injection of anti-VEGF therapy, and an AH sample of approximately 0.1 mL was collected. The collected sample was immediately transferred to a prelabelled 1-mL EP tube and stored in a −80 °C ultralow-temperature freezer.

An EV Array chip platform was used to detect EV membrane proteins in AH samples. This chip contained 435 target antibodies, with each target detected in duplicate. Each well was first loaded with 100 μL of blocking solution (3% BSA) and incubated at room temperature for 30 min. The blocking solution was then removed. The AH sample was diluted to 100 μL with PBS, added to the chip, and incubated at room temperature for 60 min. After the reaction mixture was removed, the chip was washed three times with PBST (0.05% Tween 20). Two hundred microlitres of biotinylated detection antibody (anti-human CD9/CD63/CD81; LifeSpan BioSciences, Seattle, WA, United States). was added, and the samples were incubated at room temperature for 30 min. After this step, the reaction mixture was removed, and the samples were washed three times with PBST. Subsequently, a streptavidin-conjugated Cy3 fluorescent probe (SA1010; Invitrogen, Carlsbad, California, United States) was added to each well and incubated in the dark for 30 min. The reaction mixture was removed, and the samples were washed three times with PBST. Finally, after the microarray had dried, the fluorescence signals were acquired using a 532-nm laser scanner, and the fluorescence intensity values were extracted using GenePix Pro software. The median value of the two repeat antibody spots was calculated, and the PBS antibody spot reading (background value) was subtracted from this well to obtain the antibody spot reading. After normalisation, the batch effects between the chips were analysed. The corrected values represent the absolute expression levels. Simultaneously, the geometric mean of CD9, CD63, and CD81 was used as the internal reference normalisation factor. After correction, the relative expression level was obtained.

### 2.4 Data analysis

#### 2.4.1 Bioinformatics analysis

Gene Ontology (GO) and Kyoto Encyclopaedia of Genes and Genomes (KEGG) pathway analyses were performed using the clusterProfiler package in R software (V 4.4.3), with visualisations generated via the online platform (https://www.chiplot.online/) to elucidate gene functions and disease mechanisms. To investigate the associations among the differentially expressed proteins across the comparison groups, protein interaction networks were constructed using the STRING database (https://string-db.org/) and Cytoscape (V 3.10.3). The threshold for screening was set at a combined score >0.4. To analyse key pathways and genes involved in DR- and RVO-induced macular edema, GSEA enrichment analysis and visualisation were performed using GSEA software (V 4.4.0) (Subramanian et al., 2005).

Cell-of-origin analysis utilized publicly available retinal single-cell RNA sequencing data. We downloaded the GSE245561 dataset from a public database to investigate the potential cellular origins of EV membrane proteins that differ between treatment-naive and refractory diabetic macular edema (DME). In contrast, the GSE230348 dataset was used for subsequent analysis of retinal vein occlusion-associated macular edema (RVO-ME). All bioinformatics analyses were performed using R software (version 4.4.3). Single-cell RNA sequencing analyses were conducted using Seurat (version 5.3.0). Quality control retained cells with more than 200 detected genes, fewer than 6,000 detected genes, and mitochondrial gene percentages below 20%. Principal component analysis, graph-based clustering (resolution = 0.8), and UMAP dimensional reduction were performed using default Seurat workflows unless otherwise specified. Cell clusters were annotated based on classic marker genes. Genes encoding EV membrane proteins were then mapped to the single-cell transcriptomic dataset. The AverageExpression function in Seurat was used to compute the average gene expression for each annotated cell type. Heatmaps and violin plots were generated to visualize the expression distribution of the corresponding EVs membrane protein-associated genes across different cell populations. Cell types exhibiting relatively enriched transcriptional expression were considered potential cellular sources of the corresponding EVs’ membrane proteins. The original analysis scripts are available from the corresponding author upon reasonable request for academic purposes.

#### 2.4.2 Statistical analysis

SPSS version 26.0 (SPSS Inc., Chicago, IL, United States) was used. Continuous variables are reported as the mean ± standard deviation. In terms of data type and distribution, independent-samples t tests were used for normally distributed continuous variables, whereas Mann–Whitney U tests were used for non-normally distributed continuous variables. Chi-square tests were used for categorical variables. Correlation analysis was performed using either the Pearson method or the Spearman method, depending on the data distribution characteristics. Raw data from the EV Array were analysed with SPSS to identify differential expression; Prism 9 (V 9.5.1) was used to visualise the differential expression in EVs and generate ROC curves. Volcano plots and heatmaps for comparisons were produced using R (V 4.4.3) and other packages (“limma”, “ggplot2”, “heatmap”, etc.). *P* values <0.05 were considered to indicate statistical significance.

## 3 Results

### 3.1 Demographic characteristics of the study population

**FIGURE 2:**
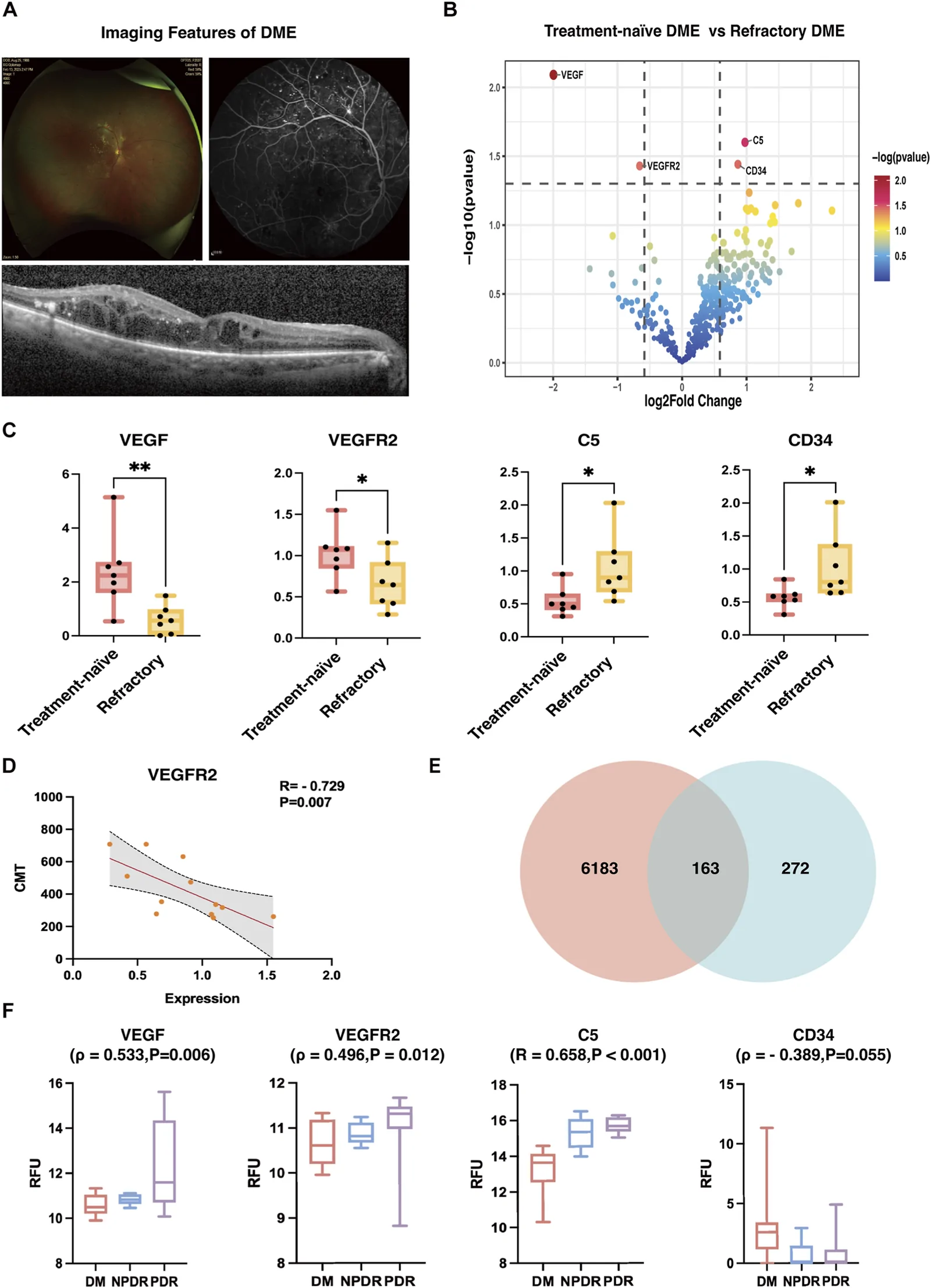
Differential expression of aqueous humour EV membrane proteins in patients with treatment-naïve and refractory DME. **(A)** Representative fundus photographs of patients with DME, showing ME and structural alterations. **(B)** Volcano plot of differentially expressed EV membrane proteins between patients with treatment-naïve DME and those with refractory DME. **(C)** Expression levels of the four candidate membrane proteins across patient cohorts. Box plots show the distribution for each group. **(D)** Correlations of membrane protein expression with CMT. VEGFR2 levels are inversely correlated with CMT. **(E)** Overlap between the differentially expressed EV membrane proteins identified in this study and AH proteins. **(F)** Validation of candidate protein expression levels across diabetic retinopathy severity grades using independent public proteomic datasets. *P* < 0.05 was considered to indicate statistical significance. ns = *P* > 0.05; *P < 0.05; **P < 0.01; ***P < 0.001; ****P < 0.0001. ME, macular edema; AH, aqueous humour; EV, extracellular vesicle; DME, diabetic macular edema; RFU, relative fluorescence units; CMT, central macular thickness.

This study included 28 patients with treatment-naïve and refractory DME and RVO-ME (Figure 2A; Figure 4A). The clinical indicators collected in this work included sex; age; duration of diabetes; and levels of glycated haemoglobin (HbA1c), total cholesterol (TC), triglyceride (TG), high-density lipoprotein (HDL), and low-density lipoprotein (LDL).

There were no significant differences in any clinical indicators between the treatment-naïve and refractory DME groups (Table 1). Statistically significant differences in diastolic blood pressure (*P* = 0.018), mean arterial pressure (*P* = 0.011), and serum ALB concentration (*P* = 0.029) were detected between the treatment-naïve and refractory RVO-ME groups (Table 2).

**TABLE 1:** Characteristic of subjects for DME Aqueous Humor collection.

Variable	Treatment-naïve DME	Refractory DME	*P*
Sex			
Male	5(71.4%)	5(71.4%)	1.000^b^
Female	2(28.6%)	2(28.6%)	
Age, y	60.71±8.770	59.71±9.160	0.838^a^
Diabetes Duration, y	13.86±4.337	11.14±9.634	0.364^c^
Systolic Pressure, mmHg	129.29±21.077	147.71±14.660	0.082^a^
Diastolic Pressure, mmHg	75.43±11.942	80.57±10.830	0.415^a^
Mean Arterial Pressure, mmHg	93.38±11.924	102.95±11.112	0.146^a^
FPG	7.03±1.986	8.23±1.853	0.317^a^
Hba1c	8.47±1.859	7.68±0.928	0.368^a^
Total protein, g/L	72.59±3.769	74.07±5.757	0.578^a^
Albumin, g/L	44.07±2.296	43.23±2.247	0.501^a^
Globulin,g/L	28.51±3.737	30.84±4.177	0.293^a^
Globulin Ratio,	1.57±0.233	1.42±0.168	0.192^a^
Blood Urea Nitrogen, mg/dl	7.92±3.344	7.09±2.215	0.594^a^
Blood Creatinine, μmol/L	82.57±20.040	84.14±25.419	0.900^a^
Urea Nitrogen/Creatinine, μmol/L	0.10±0.032	0.09±0.030	0.550^a^
Cholesterol, mmol/L	4.68±1.186	3.94±1.075	0.391^c^
Triglyceride, mmol/L	1.29±0.413	1.40±0.617	0.830^a^
High Density Lipoprotein, mmol/L	1.45±0.629	1.24±0.357	0.250^c^
Low Density Lipoprotein, mmol/L	2.39±0.613	1.98±0.747	0.301^a^

DME = Diabetic Macular Edema; FP = Fasting Plasma Glucose; SD = Standard Deviation; Independent samples t-tests were used for variables that were normally distributed with homogeneity of variance; otherwise, the Mann–Whitney U test was applied. For non-normally distributed samples with heterogeneity of variance, the rank-sum test was employed for difference analysis. *P* < 0.05 is considered statistically significant.

^a^Independent samples t-tests; ^b^x^2^ test. ^c^Mann–Whitney U test was applied.

**TABLE 2:** Characteristic of subjects for RVO aqueous humor collection.

Variable	Treatment-naïve RVO-ME	Refractory RVO-ME	P
Sex			
Male	3(42.9%)	3(42.9%)	1.00^b^
Female	4(57.1%)	4(57.1%)	
Age, y	63.14±5.273	61.86±9.118	0.752^a^
Hypertension Duration y	2.00±4.472	7.43±6.925	0.062^c^
Systolic Pressure, mmHg	142.43±7.413	133.00±10.312	0.073^a^
Diastolic Pressure, mmHg	83.29±6.626	73.86±6.230	**0.018** ^a^
Mean Arterial Pressure, mmHg	103.00±6.610	93.57±5.081	**0.011** ^a^
FPG	5.20±0.851	6.12±1.316	0.144^a^
Hba1c	NA	6.70±0.500	NA
Total Protein, g/L	72.26±2.367	71.06±4.295	0.530^a^
Albumin, g/L	44.07±1.008	42.79±0.853	**0.029** ^a^
Globulin, g/L	28.21±1.906	28.27±4.501	0.976^a^
Globulin Ratio	1.57±0.101	1.55±0.243	0.844^a^
Blood Urea Nitrogen, mg/dl	4.36±0.807	5.17±1.703	0.338^c^
Blood Creatinine, μmol/L	60.71±10.996	65.57±5.884	0.323^a^
Urea Nitrogen/Creatinine μmol/L	0.08±0.015	0.08±0.034	0.948^c^
Cholesterol, mmol/L	4.52±1.129	4.65±0.811	0.839^a^
Triglyceride, mmol/L	1.63±0.583	1.52±0.419	0.719^a^
High Density Lipoprotein, mmol/L	1.26±0.303	1.21±0.226	0.807^c^
Low Density Lipoprotein, mmol/L	2.28±0.673	2.41±0.698	0.753^a^

RVO-ME = Retinal Vein Occlusion-related Macular Edema; FP = Fasting Plasma Glucose; SD = Standard Deviation; Independent samples t-tests were used for variables that were normally distributed with homogeneity of variance; otherwise, the Mann–Whitney U test was applied. For non-normally distributed samples with heterogeneity of variance, the rank-sum test was employed for difference analysis. P < 0.05 is considered statistically significant.

The bold values indicate P < 0.05.

^a^Independent samples t-tests; ^b^x^2^ test. ^c^Mann–Whitney U test was applied.

### 3.2 Differential EV membrane protein profiles in treatment-naïve and refractory DME

In this study, a total of 435 EV membrane proteins were detected. Four differentially expressed membrane proteins were identified between patients with treatment-naïve DME and those with refractory DME (Figure 2B). VEGF (*P* = 0.008, FC = 3.985) and VEGFR2 (*P* = 0.037, FC = 1.578) were significantly more highly expressed in patients with treatment-naïve DME than in those with refractory DME. Conversely, C5 (*P* = 0.025; FC = 0.509) and CD34 (*P* = 0.036; FC = 0.549) were more highly expressed in patients with refractory DME (Figure 2C). Furthermore, correlation analysis within the DME group showed that VEGFR2 expression was significantly inversely correlated with CMT (R = −0.729; P = 0.007) (Figure 2D).

To confirm that the differentially expressed membrane proteins identified in this study were not incidental, we conducted a comparative analysis using publicly available AH proteomics databases. Proteomic data from patients with diabetes mellitus (DM), nonproliferative diabetic retinopathy (NPDR), and proliferative diabetic retinopathy (PDR), reported by Julian W et al., revealed a total of 6,346 proteins (Wolf et al., 2023), of which 163 overlapped with the protein spectrum in this study (Figure 2E). Notably, VEGF, VEGFR2, C5, and CD34, identified in our study as significant differentially expressed membrane proteins, also showed significant differential expression in this external study. Further correlation analysis revealed that all these proteins except CD34, i.e., VEGF (*ρ* = 0.533; *P* = 0.006), VEGFR2 (*ρ* = 0.496; *P* = 0.012), and C5 (*R* = 0.658; *P* < 0.001), were significantly positively correlated with the severity of DR (Figure 2F).

### 3.3 Cell-of-origin mapping of differentially expressed EV membrane proteins in DME

**FIGURE 3:**
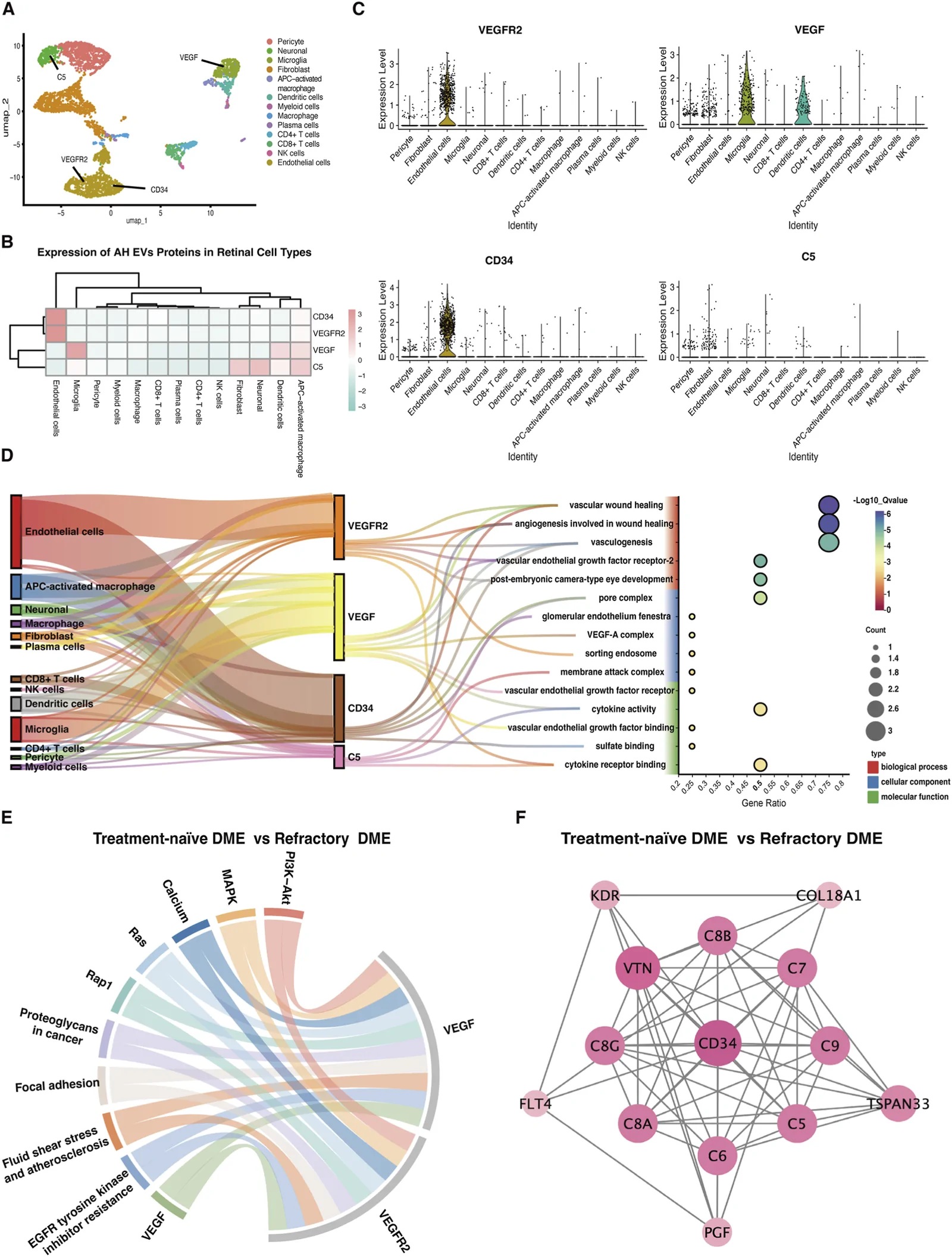
Cellular origins and functional implications of differentially expressed EV membrane proteins in DME. **(A)** UMAP of single-cell transcriptomic data from diabetic fibrovascular membranes, colour-coded by major retinal cell types. **(B)** Heatmap depicting the cell type-specific expression patterns of the four candidate membrane proteins. **(C)** Violin plots comparing the expression levels of the four membrane proteins across the identified cell subtypes. **(D)** Sankey diagram linking protein expression to cell origins and associated biological processes, with edge weight representing expression strength. **(E)** Significantly enriched KEGG pathways among proteins elevated in refractory DME. **(F)** The protein–protein interaction network was centred on CD34, highlighting its role as a key hub protein. EV, extracellular vesicle; DME, diabetic macular edema.

Tracing analysis of the four differentially expressed membrane proteins in the DME group revealed their primary sources: endothelial cells, APC-activated macrophages, neuronal cells, macrophages, fibroblasts, plasma cells, CD8^+^ T cells, NK cells, dendritic cells, microglia, CD4^+^ T cells, pericytes, and myeloid cells (Figures 3A–C).

Subsequent GO enrichment analysis of the differentially expressed membrane proteins revealed that these alterations were primarily involved in the biological process of vascular wound healing. Cellular components were enriched in pore complexes, whereas molecular functions were enriched in vascular endothelial growth factor receptors (Figure 3D). KEGG enrichment analysis revealed that the differentially expressed membrane proteins that were highly expressed in AH from treatment-naïve DME patients were concentrated in 10 key pathways (Figure 3E), with the VEGF signalling pathway being the most prominent pathway.

In this study, the STRING database was used to construct a protein–protein interaction network, thereby identifying disease-associated core genes and their regulatory pathways. In the DME group, four differentially expressed proteins were identified between treatment-naïve DME and refractory DME. We expanded the scope of the search to include indirectly associated proteins. Among the differentially expressed membrane proteins, C5, CD34, and KDR (VEGF) interacted with other proteins (75%) (Figure 3F).

### 3.4 Differences in EV membrane protein alterations between treatment-naïve and refractory RVO-ME

**FIGURE 4:**
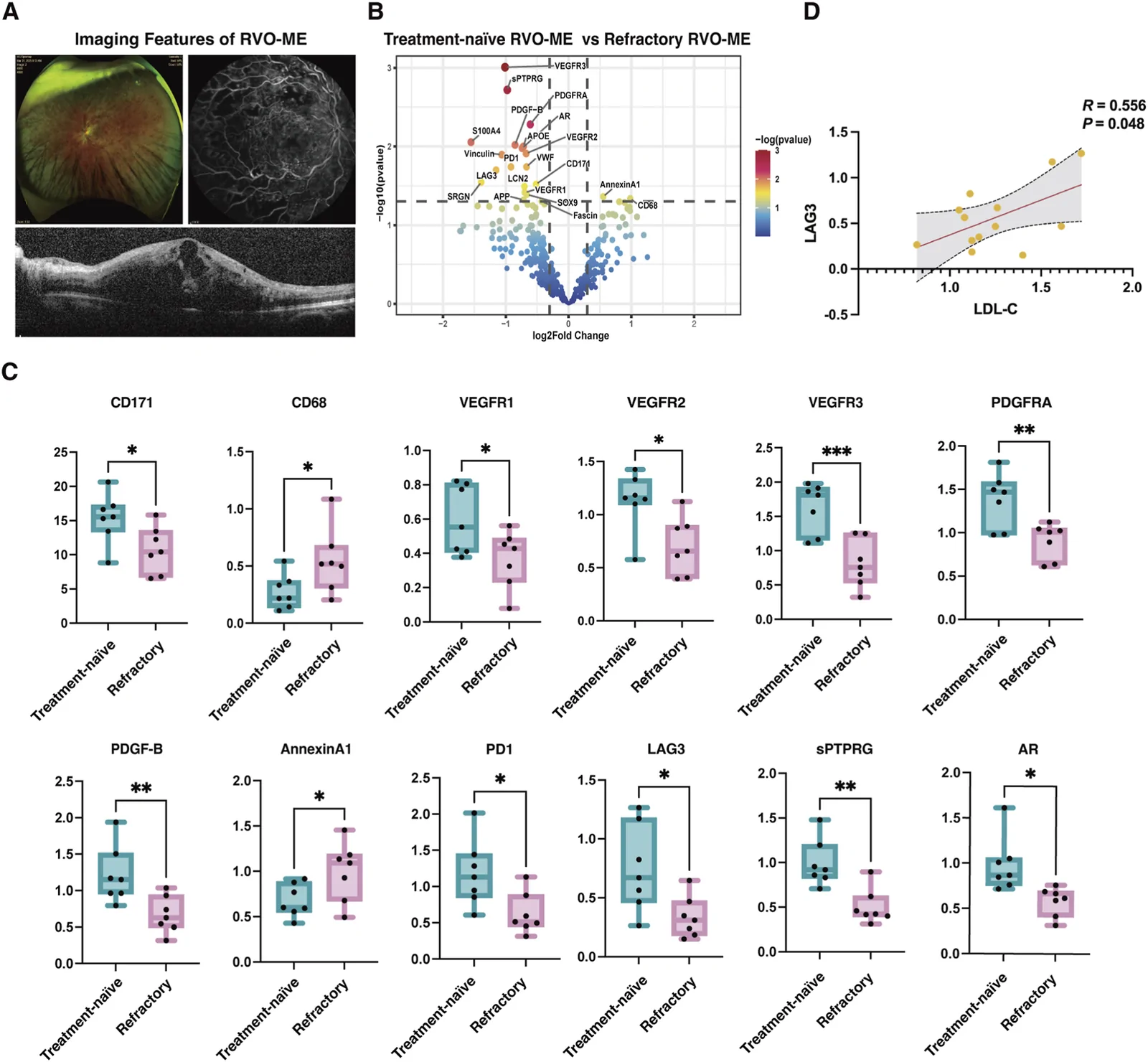
Differential EV membrane protein expression between patients with treatment-naïve RVO-ME and those with refractory RVO-ME. **(A)** Representative fundus images of RVO-ME, demonstrating ME and structural alterations. **(B)** Volcano plot identifying differentially expressed EV membrane proteins between patients with treatment-naïve RVO-ME and those with refractory RVO-ME. **(C)** Expression levels of selected differentially expressed membrane proteins across patient groups. A total of 21 proteins were significantly altered. **(D)** Correlations between LAG3 expression and HDL-C levels. EV, extracellular vesicle; RVO-ME, retinal vein occlusion-related macular edema; HDL-C, high-density lipoprotein cholesterol.

A comparison of the treatment-naïve RVO-ME and refractory RVO-ME groups revealed 21 differentially expressed membrane proteins (Figure 4B; Supplementary Figure S1). Categorisation of their properties revealed that CD molecules exhibited opposite expression trends between groups: the expression of the cell adhesion molecule CD171 was significantly upregulated in treatment-naïve RVO-ME (*P* = 0.030, FC = 1.430), whereas the expression of the macrophage marker CD68 (*P* = 0.046, FC = 0.506) was more strongly upregulated in refractory RVO-ME (Figure 4C). Additionally, the expression of VEGF family proteins and other differentially expressed membrane proteins, such as VEGFR1 (*P* = 0.038, FC = 1.623); VEGFR2 (*P* = 0.012, FC = 1.599); VEGFR3 (*P* = 0.001, FC = 2.017); the vasculature-associated proteins PDGFRA (*P* = 0.005, FC = 1.526) and PDGF-B (*P* = 0.010, FC = 1.806); the inflammation- and immunity-related proteins PD1 (*P* = 0.018, FC = 1.890), LAG3 (*P* = 0.020, FC = 2.226); and other membrane proteins, was upregulated in the AH of patients with treatment-naïve RVO-ME (Figure 4C).

Additionally, in RVO-ME, the differential membrane protein LAG3 was significantly positively correlated with high-density lipoprotein (Figure 4D).

### 3.5 Cell-of-origin mapping of differentially expressed EV membrane proteins in RVO-ME

**FIGURE 5:**
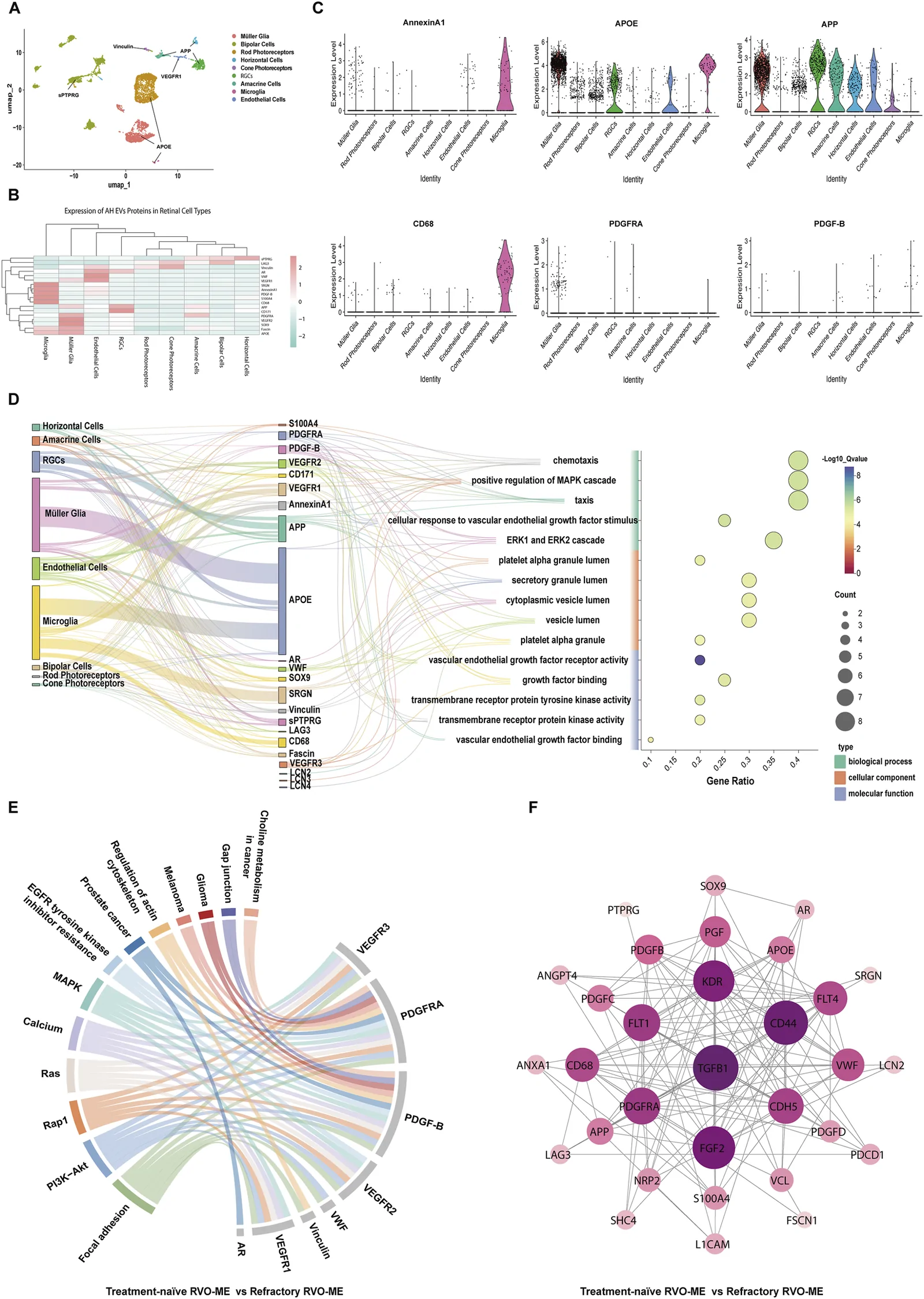
Cellular origins and functional networks of differentially expressed EV membrane proteins in RVO-ME. **(A)** UMAP visualisation of single-cell RNA sequencing data from normal human retinas, coloured according to distinct retinal cell populations. **(B)** Heatmap displaying cell-type-specific expression patterns of selected differentially expressed membrane proteins. **(C)** Violin plots showing the expression levels of selected proteins across major retinal cell types. **(D)** Sankey diagram integrating protein–cell associations and enriched biological processes from Gene Ontology analysis. **(E)** KEGG pathway enrichment analysis of proteins differentially expressed between treatment-naïve RVO-ME and refractory RVO-ME. **(F)** Protein–protein interaction network with TGFβ1 as the central hub protein. EV, extracellular vesicle; RVO-ME, retinal vein occlusion-related macular edema.

Tracing analysis found that 21 differentially expressed membrane proteins in the RVO-ME group primarily originate from retinal cells, including horizontal cells, cone photoreceptors, endothelial cells, RGCs, bipolar cells, rod photoreceptors, Müller glia, and microglia (Figures 5A–C).

In addition, GO enrichment analysis revealed that the differentially expressed membrane proteins were involved primarily in the biological process of vascular endothelial growth factor signalling. Cellular components were enriched in platelet alpha granules, and molecular functions were enriched in vascular endothelial growth factor receptors (Figure 5D). KEGG enrichment analysis of the differentially expressed membrane proteins revealed 13 pathways (Figure 5E), including multiple core pathways related to cellular dynamics and survival. These pathways included focal adhesion; regulation of the actin cytoskeleton; gap junctions; and key pro-survival and proliferation signalling pathways, such as the PI3K–Akt signalling pathway.

In the RVO-ME group, 18 of the 21 differentially expressed proteins interacted with other proteins (86%), suggesting a multidimensional synergistic pathological mechanism in refractory RVO-ME (Figure 5F). The core proteins in the PPI network included CD44, CD68, KDR (VEGF), FLT1 (VEGFR1), VCL (vinculin), S100A4, and APOE.

### 3.6 Cross-disease differential expression between DME and RVO-ME

**FIGURE 6:**
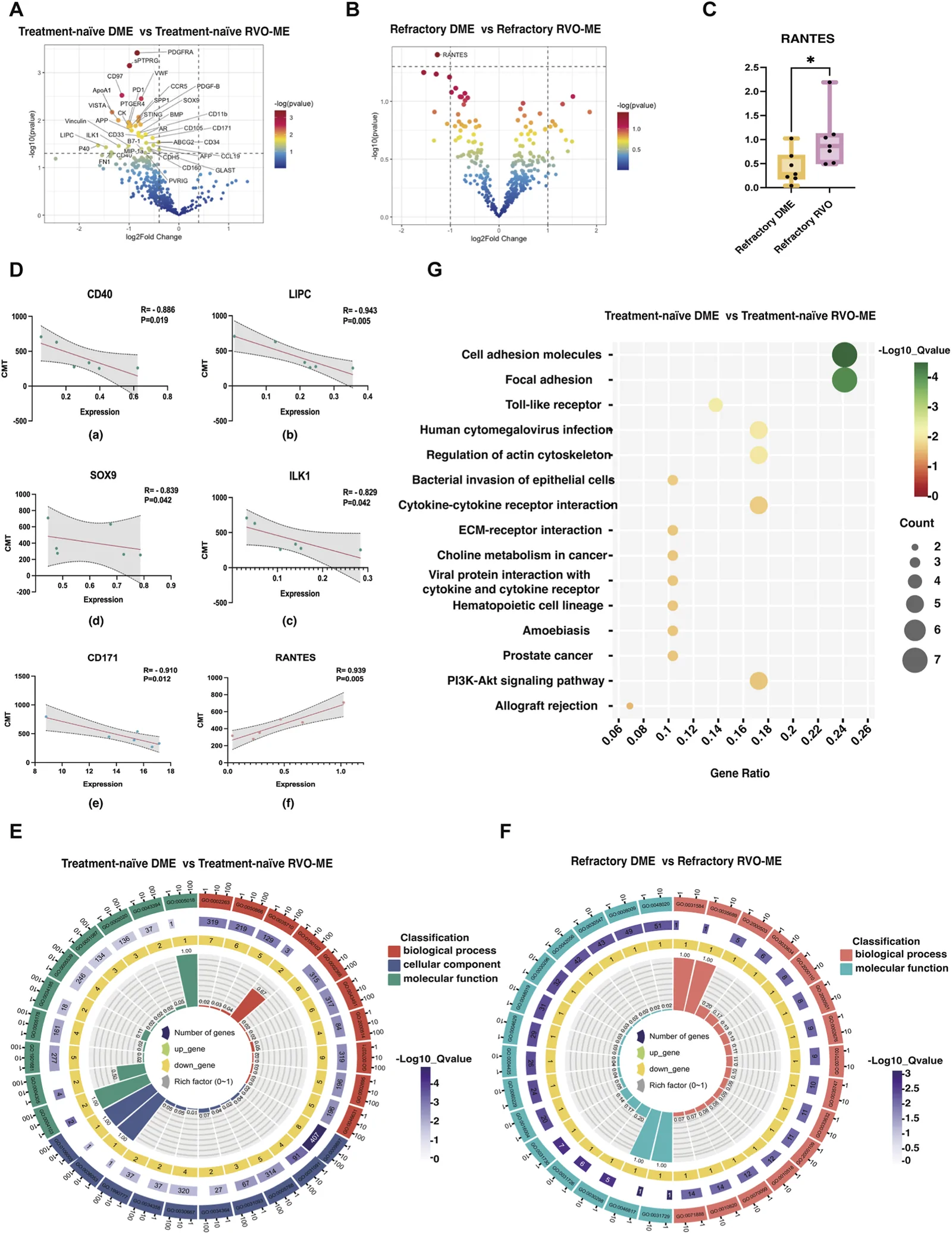
Comparative proteomic profiling of EV membrane proteins in DME and RVO-ME. **(A)** Volcano plot of differentially expressed EV membrane proteins between treatment-naïve DME and RVO-ME patients. **(B)** Volcano plot of differentially expressed EV membrane proteins between refractory DME and RVO-ME. **(C)** Expression level of RANTES, the sole differentially expressed membrane protein identified between refractory DME and RVO-ME. **(D)** Correlations between key membrane protein levels and CMT across disease stages and subtypes. **(E)** GO functional enrichment analysis of proteins differentially expressed between treatment-naïve DME and RVO-ME. **(F)** GO functional enrichment analysis of proteins differentially expressed between refractory DME and RVO-ME. **(G)** KEGG pathway enrichment analysis of proteins differentially expressed between treatment-naïve DME and RVO-ME. EV, extracellular vesicle; DME, diabetic macular edema; RVO-ME, retinal vein occlusion–related macular edema; CMT, central macular thickness.

We further investigated whether significant differences in the EV membrane proteome were present between different diseases (DME and RVO-ME). The results revealed 34 differentially expressed membrane proteins between treatment-naïve DME and treatment-naïve RVO-ME (Figure 6A; Supplementary Figure S1). All were downregulated in treatment-naïve DME (Figure 6A). The expression of multiple CD molecules, namely, CD33 (*P* = 0.012; FC = 0.571), CD34 (*P* = 0.030; FC = 0.634), CD40 (*P* = 0.041; FC = 0.487), CD97 (*P* = 0.003; FC = 0.452), CD105 (*P* = 0.023; FC = 0.597), and CD171 (*P* = 0.024; FC = 0.693), was downregulated in the treatment-naïve DME group (Supplementary Figure S1). In contrast, only one membrane protein was significantly differentially expressed between refractory DME and refractory RVO-ME: RANTES (*P* = 0.039, FC = 0.415) (Figure 6B), with downregulated expression in refractory DME (Figure 6C).

GO enrichment analysis revealed that membrane proteins with biological functions primarily involved in immune cell activation and regulation were significantly enriched in the treatment-naïve RVO-ME group compared with the treatment-naïve DME group. These proteins were notably enriched in immunity-related pathways, such as cell activation involved in the immune response, CD4 positivity, alpha/beta T-cell activation, and negative regulation of monocyte activation. They were also enriched in related signalling pathways, such as phosphatidylinositol 3-kinase/protein kinase B signalling and the ERK1 and ERK2 cascades. Cellular components were enriched in multiple important cellular structures and functional regions, such as the external side of the plasma membrane. The enriched molecular functions were primarily related to choline kinase activity (Figure 6E). The membrane protein RANTES, differentially expressed between the refractory DME group and the refractory RVO-ME group, was primarily involved in the biological function “activation of phospholipase D activity” and the molecular function “CCR4 chemokine receptor binding” (Figure 6F). KEGG enrichment analysis revealed that in the treatment-naïve DME group vs. the treatment-naïve RVO-ME group, the pathways with the greatest differential membrane protein abundance were cell adhesion molecules and focal adhesion. These pathways were not only the most significantly enriched but also highly abundant, suggesting that the primary differences between treatment-naïve DME and RVO-ME were related to key processes such as cell adhesion, extracellular matrix attachment, and endothelial barrier regulation (Figure 6G). The membrane protein RANTES was significantly enriched in the Toll-like receptor signalling pathway, TNF signalling pathway, and chemokine signalling pathway, suggesting that refractory ME was involved in the activation of innate immunity and proinflammatory responses (Supplementary Figure S2).

Additionally, in this study, PPI networks for differential membrane proteins across disease states at the same stage spanning different diseases were constructed to explore shared pathological mechanisms and disease-specific pathways between DME and RVO. Among the 34 differential membrane proteins in treatment-naïve DME vs. treatment-naïve RVO-ME, 32 (94.1%) interacted with other proteins. The core proteins included FN1, VWF, ITGAM (CD11b), PDGFRA, PDGFB, APOA1, PDCD1 (PD1), and ENG (CD105) (Supplementary Figure S2).

### 3.7 Associations between EV membrane proteins and clinical parameters

In the correlation analysis between CMT and differentially expressed membrane proteins in treatment-naïve DME and treatment-naïve RVO-ME, it was observed that CD40, LIPC, ILK1, and SOX9 in the AH of treatment-naïve DME patients were significantly negatively correlated with CMT (Figure 6D), whereas only CD171 in treatment-naïve RVO-ME was significantly negatively correlated with CMT (*R* = −0.910, *P* = 0.012) (Figure 6D). In the differential membrane protein analysis between refractory DME patients and refractory RVO-ME patients, only the level of RANTES in the AH of refractory DME patients was significantly positively correlated with CMT (Figure 6D).

### 3.8 GSEA enrichment signatures and diagnostic potential of EV membrane proteins

**FIGURE 7:**
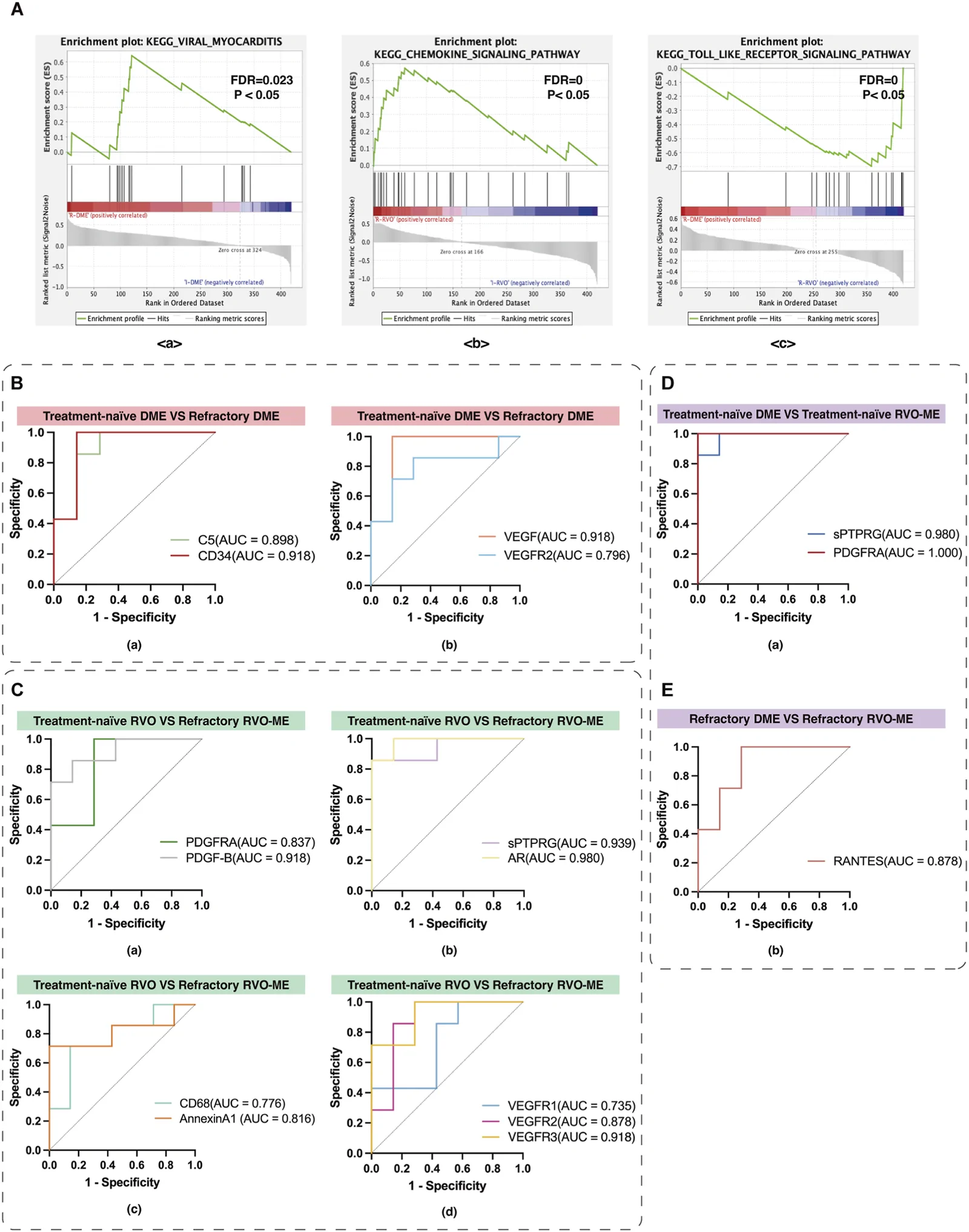
Enrichment signatures and diagnostic potential of EV membrane protein profiles. **(A) (a)** Gene set enrichment analysis (GSEA) of membrane proteins differentiating treatment-naïve and refractory DME. **(b)** GSEA of membrane proteins differentiating treatment-naïve and refractory RVO-ME patients. **(c)** GSEA of membrane proteins differentiating treatment-naïve DME and RVO-ME patients. **(B)** ROC curves assessing the diagnostic capacity of four candidate proteins to discriminate treatment-naïve from refractory DME. **(C)** Diagnostic performance of differential membrane proteins in RVO-ME: **(a)** top two proteins by AUC; **(b)** PDGF-related proteins; **(c)** inflammation- and immunity-related proteins; **(d)** VEGF-related proteins. **(D)** ROC curves for the top two membrane proteins discriminating treatment-naïve DME from treatment-naïve RVO-ME. **(E)** ROC curves for the top two membrane proteins discriminating refractory DME from refractory RVO-ME. P < 0.05 and FDR <0.05 were considered to indicate statistical significance. EV, extracellular vesicle; DME, diabetic macular edema; GSEA, gene set enrichment analysis; RVO-ME, retinal vein occlusion-related macular edema; AUC, area under the curve; ROC, receiver operating characteristic.

We next performed GSEA to examine the global enrichment patterns of differentially expressed protein-coding genes across the comparison groups. Notably, relative to the treatment-naïve DME group, the refractory DME group showed significant overall enrichment of EV membrane proteins in the KEGG_VIRAL_MYOCARDITIS pathway (FDR = 0.023, *P* < 0.05). Although this pathway is labelled “viral myocarditis”, the genes that make up this pathway include a wide range of cytokines, chemokines and immune effector molecules. Many of these are not specific to viral infection, but instead represent a general transcriptional signature of innate immune activation and inflammatory tissue injury. For RVO-ME, refractory samples exhibited marked enrichment of the chemokine signaling pathway compared with treatment-naïve ones (FDR = 0, *P* < 0.05) (Figure 7A), indicating heightened activity of chemokine-related genes in refractory RVO-ME. When we directly compared the refractory stages, the toll-like receptor signaling pathway was significantly enriched in refractory RVO-ME (FDR = 0, *P* < 0.05) (Figure 7A), whereas this enrichment was not observed in refractory DME. These distinct pathway activation patterns suggest disease-specific molecular signatures that may hold diagnostic or prognostic value.

To validate the diagnostic value of the differentially expressed EV membrane proteins, we performed ROC analysis on all such proteins within each group. In the DME group, CD34 and VEGF yielded the highest AUCs (0.918 each), followed by C5 (0.898) and VEGFR2 (0.796) (Figure 7B), all showing good discriminatory power for refractory DME. Similarly, the 21 proteins in the RVO-ME group demonstrated high diagnostic performance for refractory RVO-ME, with AR (AUC = 0.980) and sPTPRG (AUC = 0.939) ranking top (Figure 7C). Across-disease analysis showed that PDGFRA had the highest AUC for distinguishing treatment-naïve DME from treatment-naïve RVO-ME (Figure 7D). RANTES, the only protein differing between the two refractory groups, achieved an AUC of 0.878, indicating strong discriminatory ability between refractory ME secondary to DR and that secondary to RVO (Figure 7E).

### 3.9 Shared pathways potentially driving progression to refractory ME

A comparison of the results of the KEGG enrichment analysis for membrane proteins with significant differences between the DME and RVO-ME groups revealed seven shared pathways with significant enrichment: focal adhesion, the PI3K–Akt signalling pathway, the Rap1 signalling pathway, the Ras signalling pathway, the calcium signalling pathway, the MAPK signalling pathway, and EGFR tyrosine kinase inhibitor resistance. These pathways collectively indicated dysregulation of core biological processes, including cell proliferation, survival, migration, and angiogenesis (Supplementary Table S3).

## 4 Discussion

Macular edema is among the most clinically significant complications of DR and RVO, substantially impairing patients’ quality of life through central vision loss, visual distortion, and compromised stereoscopic vision (Hayreh et al., 2011; Fenwick et al., 2012; Varma et al., 2012; Bandello et al., 2017; Morikawa et al., 2019; Assi et al., 2021; Okamoto et al., 2021; Sugiura et al., 2021; Murakami et al., 2022). Advances in understanding the retinal microstructure have revealed that prolonged or substantial fluid accumulation in the macular region not only induces degenerative changes and mechanical damage to outer retinal structures (e.g., the external limiting membrane, ellipsoid zone, and photoreceptors) (Ehlers et al., 2019; Borrelli et al., 2023; do Nascimento et al., 2024) but also may trigger irreversible neuroretinal layer impairment and aberrant glial cell activation (Lai et al., 2023). Even after edema has subsided, functional nerve damage still occurs (Guyon et al., 2017; Sadda et al., 2020; Park et al., 2024). However, responses to anti-VEGF therapy vary among patients. Approximately 30%–40% of patients exhibit an inadequate response to this treatment and present with “refractory” or “persistent” macular edema (Zhang et al., 2022). Therefore, it is crucial to explore the pathogenesis of refractory ME and identify diagnostic markers in order to manage ME effectively and select potential therapeutic targets.

As an integral component of the intraocular microenvironment, AH contains a variety of bioactive molecules, including EVs measuring approximately 30–200 nm in diameter. These EVs carry cell-specific membrane proteins that are extremely valuable for analysing disease mechanisms and developing targeted therapies (Melo et al., 2015; Chen et al., 2023; Lee et al., 2023). On the basis of membrane proteomic profiling of AH EVs, we classified ME into treatment-naïve and refractory stages to systematically investigate the molecular basis of its transition from early leakage to treatment resistance. Among the 435 identified membrane proteins, VEGF signalling was downregulated in the refractory group with anti-VEGF treatment compared with the treatment-naïve stage of ME in both diseases. C5 and CD34 were significantly more highly expressed in patients with refractory DME, whereas CD68 and Annexin A1 were more strongly upregulated in the refractory RVO-ME group.

During DME progression, GSEA indicated enhanced immune and inflammatory activation in the refractory stage. In line with these findings, we observed significantly increased expression of complement component C5 and the endothelial progenitor cell marker CD34 (Chambers et al., 2021) in the refractory group. Differential membrane protein tracing further implicated inflammatory and immune cells, suggesting a pathological shift from VEGF-driven acute leakage to chronic inflammation characterised by complement activation and endothelial cell engagement. Specifically, complement activation, particularly C5-mediated formation of the membrane attack complex, may directly promote cellular injury. Preclinical evidence has shown that blocking C5 suppresses inflammasome activation in retinal pigment epithelial cells, inhibits choroidal neovascularisation and preserves retinal neuronal function (Brandstetter et al., 2015; Gassel et al., 2021). Although C5 inhibitors such as avacincaptad pegol have been approved by regulators for the treatment of geographic atrophy, their use in the treatment of DME remains largely unexplored. Our findings provide a molecular rationale for investigating C5 inhibition as a potential adjunctive therapy in anti-VEGF-refractory DME. Moreover, the observed downregulation of VEGF and VEGFR2 in refractory DME supports the notion that repeated anti-VEGF therapy can trigger feedback inhibition of this pathway. As a result, alternative inflammatory and immune pathways are activated, ultimately contributing to suboptimal treatment outcomes.

In refractory RVO-ME, anti-VEGF therapy leads to the downregulation of VEGF and PDGF, while the macrophage/microglia markers CD68 (Holness and Simmons, 1993) and Annexin A1 are significantly upregulated and play pivotal roles in protein interaction networks. These changes suggest persistent vascular leakage and inflammatory activity, underscoring the pivotal role of chronic inflammation and innate immune cell infiltration in mediating resistance to anti-VEGF treatment. Macrophages play a crucial role in the progression of RVO (Kim et al., 2025) by secreting inflammatory factors and facilitating efferocytosis, the process by which apoptotic cells are cleared from the body. Annexin A1 acts as a key regulator of this process (Fang et al., 2025). Inflammatory cytokines not only contribute to disease pathogenesis but also may serve as biomarkers for predicting disease severity and treatment response (Minaker et al., 2020). Therefore, targeting macrophage-related inflammatory pathways represents a promising direction for future therapeutic development. Conversely, PDGF-B, an essential growth factor for pericyte and smooth muscle cell recruitment (Abramsson et al., 2003; Andrae et al., 2008; Aguilera and Brekken, 2014), contributes to vascular stabilisation (Dubrac et al., 2018). These findings suggest that while anti-VEGF therapy mitigates pathological neovascularisation, it may also suppress normal compensatory vascular repair mechanisms. Ultimately, overactivated inflammatory responses can override or inhibit these reparative signals. This phenomenon leads to sustained immune cell recruitment, amplified activation of the inflammatory pathway, and disruption of tissue repair. These factors collectively drive disease progression towards the refractory state.

Our study highlights the dual role of EV proteins, which serve as both reporters of cellular activity and promising diagnostic biomarkers. Specifically, C5 and CD34 have emerged as potential molecular markers for the diagnosis of refractory DME. Furthermore, the involvement of macrophages and APs indicates that RVO requires a greater focus on anti-inflammatory strategies in addition to conventional anti-VEGF therapy. During the refractory phase of both diseases, we identified the chemokine RANTES as the only differentially expressed membrane protein in EVs. RANTES expression is significant in the inner retina (Meleth et al., 2005; Duncan et al., 2017) and is closely related to the phenotypes of Müller cells and retinal ganglion cells (Duncan et al., 2017; Duncan et al., 2018). Müller cells are known to be the core glial cells that maintain retinal homeostasis (Lai et al., 2023). Their dysfunction, which includes osmotic swelling and decreased fluid clearance caused by Kir4.1 channel downregulation, plays a vital role in the development and persistence of ME (Pannicke et al., 2006; Reichenbach et al., 2007). Previous studies have also shown that the expression of RANTES is significantly upregulated in DR (Meleth et al., 2005; Midena et al., 2019). However, the present study revealed that RVO was upregulated to a greater extent than DME. As an essential inflammatory chemokine, RANTES can recruit and activate inflammatory cells, amplifying the local inflammatory response. It can also cooperate with VEGF and other factors to disrupt vascular homeostasis, thereby promoting disease progression to a refractory state (Suffee et al., 2011; Ishida et al., 2012). The elevation of RANTES in the AH provides a molecular cornerstone for the treatment-resistant phenotype in the ME. As communication tools (Takahashi and Takakura, 2023; Kumar et al., 2024), EVs transmit signals that promote inflammatory responses and the infiltration of immune cells between cells, creating a vicious cycle. However, current treatments do not adequately address this issue (Wallsh and Gallemore, 2021). Consequently, these components represent not only promising biomarkers for disease stratification but also compelling therapeutic targets for breaking the cycle of chronic inflammation in recalcitrant cases.

Further analysis revealed that the focal adhesion pathway was significantly upregulated in both DME and RVO-ME at the initial stage. This phenomenon might indicate a compensatory response by endothelial cells and the RPE to cope with microenvironmental stress, enhancing cell–matrix adhesion to maintain barrier integrity (George et al., 2021; Aman and Margadant, 2023). Notably, activation of this pathway was greater in patients with treatment-naïve RVO-ME than in patients with DME. These findings suggest that RVO-ME results in more active integrin-mediated vascular remodelling in the early stages, whereas DME is characterised by endothelial dysfunction caused by high glucose metabolism and increased permeability driven by VEGF/inflammation. Although both diseases that cause ME are upregulated in immune and inflammatory pathways, DME favours endothelial immune regulation, whereas RVO preferentially affects chemokines and inflammatory cell-mediated pathways.

The high discriminative performance of the differentially expressed EV membrane proteins identified in this study (AUC 0.898–0.980) suggests their potential utility as feature inputs for supervised learning models, especially given the current lack of objective biomarkers to distinguish treatment-naïve from refractory macular edema prior to anti-VEGF therapy. Our proteomic dataset, obtained through minimally invasive aqueous humour sampling, offers a biologically validated molecular feature panel that could be combined with OCT parameters and systemic clinical indices to build multimodal AI classifiers. For instance, C5/CD34 in DME and CD68/Annexin A1 in RVO-ME could serve as candidate features for feature selection and model training in larger future cohorts. The consistent upregulation of RANTES across refractory disease stages points to a shared inflammatory axis that an AI model might leverage to generate a cross-disease refractoriness score. While the present study does not implement a full AI system, it provides a biologically prioritized protein feature set that, following external validation in independent cohorts, could accelerate the development of AI guided personalized treatment strategies for macular edema.

Our study has several limitations. First, the sample size was small, and future cohort expansion and longitudinal follow-up are needed to confirm the dynamic changes in protein expression. The ROC analyses presented herein are therefore single-protein evaluations with exploratory intent, rather than definitive predictive classifiers, and all candidate biomarkers require external validation in larger independent cohorts before any clinical or computational application can be developed. Second, the analysis of protein–protein interaction networks was based on public databases, and some hub proteins were not differentially expressed in this study, which might be due to their temporal expression or tissue specificity. Therefore, the results were mainly used to derive functional implications. Third, the cell-of-origin tracing analysis, while informative, relies on transcriptomic data and is therefore subject to limitations, as mRNA levels do not always reflect protein expression or selective packaging into EVs. Additionally, our findings were validated solely using public protein databases, without experimental confirmation of the differentially expressed membrane proteins. Future work will focus on expanding the cohort, performing functional validation of candidate proteins, and conducting multi-centre collaborative studies to confirm our findings.

In summary, this exploratory proteomic study identifies distinct EV membrane protein signatures associated with refractory DME and RVO-ME. Refractory DME is characterized by upregulation of complement component C5 and endothelial progenitor marker CD34, suggesting a shift toward complement-mediated and endothelial-driven inflammatory pathways. In contrast, refractory RVO-ME features upregulation of the macrophage/microglia marker CD68 and downregulation of PDGF signaling components, pointing to chronic inflammation and impaired vascular repair. These findings provide a molecular foundation for stratifying refractory ME by underlying pathophysiology and generate testable hypotheses for future mechanistic and therapeutic studies.

## Data Availability

The datasets presented in this article are not readily available because of the research team’s ongoing requirements for other studies. Requests to access the datasets should be directed to the corresponding author.
