# Supplementary material for: Aqueous humour extracellular vesicle membrane protein profiling reveals pathological features of refractory macular edema
Source: Front Cell Dev Biol. 2026 Sep 1;14:1880885. doi: 10.3389/fcell.2026.1880885 (PMC13574810; doi:10.3389/fcell.2026.1880885)
Supplement: Supplementary file 1 [file Supplementaryfile1.docx]

**Table S1. Characteristic of subjects for treatment-naïve diabetic macular edema and treatment-naïve retinal vein occlusion-related macular edema aqueous humor collection.**

|  | Treatment-naïve DME | Treatment-naïve RVO | *P* |
| --- | --- | --- | --- |
| Sex |  |  |  |
| Male,No.(%) | 5（71.4%） | 3（42.9%） | 0.592**^b^** |
| Female,No.(%) | 2（28.6%） | 4（57.1%） |  |
| Age,mean(SD),y | 60.71±8.770 | 63.14±5.273 | 0.544**^a^** |
| Hypertension Duration,mean(SD),y | 5.86±6.094 | 2.00±4.472 | 0.071**^c^** |
| Systolic Pressure,mean(SD),mmHg | 129.29±21.077 | 142.43±7.413 | 0.161**^a^** |
| Diastolic Pressure,mean(SD),mmHg | 75.43±11.942 | 83.29±6.626 | 0.161**^a^** |
| Mean Arterial Pressure,mean(SD),mmHg | 93.38±11.924 | 103.00±6.610 | 0.094**^a^** |
| FPG | 7.03±1.986 | 5.20±0.851 | **0.044^a^** |
| HbA1c | 8.47±1.859 | NA | NA |
| Total Protein,mean(SD)，g/L | 72.59±3.769 | 72.26±2.367 | 0.848**^a^** |
| Albumin,mean(SD)，g/L | 44.07±2.296 | 44.07±1.008 | 1.000**^a^** |
| Globulin,mean(SD)，g/L | 28.51±3.737 | 28.21±1.906 | 0.853**^a^** |
| Globulin Ratio,mean(SD) | 1.57±0.233 | 1.57±0.101 | 0.977**^a^** |
| Blood Urea Nitrogen,mean(SD),mg/dl | 7.92±3.344 | 4.36±0.807 | **0.018^a^** |
| Blood Creatinine,mean(SD),μmol/L | 82.57±20.040 | 60.71±10.996 | **0.026^a^** |
| Urea Nitrogen/Creatinine,mean(SD),μmol/L | 0.10±0.032 | 0.08±0.015 | 0.161**^a^** |
| Cholesterol,mean(SD),mmol/L | 4.68±1.186 | 4.52±1.129 | 0.802**^a^** |
| Triglyceride,mean(SD),mmol/L | 1.29±0.413 | 1.63±0.583 | 0.230**^a^** |
| High Density Lipoprotein,mean(SD),mmol/L | 1.45±0.629 | 1.26±0.303 | 0.485**^a^** |
| Low Density Lipoprotein,mean(SD),mmol/L | 2.39±0.613 | 2.28±0.673 | 0.752**^a^** |
| DME = Diabetic Macular Edema;RVO-ME = Retinal Vein Occlusion-related Macular Edema; FPG = Fasting Plasma Glucose;SD = Standard Deviation; Independent samples t-tests were used for variables that were normally distributed with homogeneity of variance; otherwise, the Mann–Whitney U test was applied. Chi-square tests were used for categorical variables. *P* < 0.05 is considered statistically significant.  **^a^** Independent samples t-tests; **^b^** x2 test. **^c^** Mann–Whitney U test was applied. | | | |
