# Supplementary material for: Aqueous humour extracellular vesicle membrane protein profiling reveals pathological features of refractory macular edema
Source: Front Cell Dev Biol. 2026 Sep 1;14:1880885. doi: 10.3389/fcell.2026.1880885 (PMC13574810; doi:10.3389/fcell.2026.1880885)
Supplement: Supplementary file 2 [file Supplementaryfile2.docx]

**Table S2. Characteristic of subjects for refractory diabetic macular edema and refractory retinal vein occlusion-related macular edema aqueous humor collection.**

|  | Refractory DME | Refractory RVO | *P* |
| --- | --- | --- | --- |
| Sex |  |  |  |
| Male,No.(%) | 5（71.4%） | 3（42.9%） | 0.592**^b^** |
| Female,No.(%) | 2（28.6%） | 4（57.1%） |  |
| Age,mean(SD),y | 59.71±9.160 | 63.14±5.273 | 0.669**^a^** |
| Hypertension Duration,mean(SD),y | 0.14±0.378 | 7.43±6.925 | **0.006^c^** |
| Systolic Pressure,mean(SD),mmHg | 147.71±14.660 | 133.00±10.312 | 0.051**^a^** |
| Diastolic Pressure,mean(SD),mmHg | 80.57±10.830 | 73.86±6.230 | 0.181**^a^** |
| Mean Arterial Pressure,mean(SD),mmHg | 102.95±11.112 | 93.57±5.081 | 0.065**^a^** |
| FPG | 8.23±1.853 | 6.12±1.316 | 0.086**^a^** |
| HbA1c | 7.68±0.928 | 6.70±0.500 | 0.137**^a^** |
| Total Protein,mean(SD)，g/L | 74.07±5.757 | 71.06±4.295 | 0.289**^a^** |
| Albumin,mean(SD)，g/L | 43.23±2.247 | 42.79±0.853 | 0.848**^a^** |
| Globulin,mean(SD)，g/L | 30.84±4.177 | 28.27±4.501 | 0.290**^a^** |
| Globulin Ratio,mean(SD) | 1.42±0.168 | 1.55±0.243 | 0.277**^a^** |
| Blood Urea Nitrogen,mean(SD),mg/dl | 7.09±2.215 | 5.17±1.703 | 0.064**^c^** |
| Blood Creatinine,mean(SD),μmol/L | 84.14±25.419 | 65.57±5.884 | 0.084**^a^** |
| Urea Nitrogen/Creatinine,mean(SD),μmol/L | 0.09±0.030 | 0.08±0.034 | 0.771**^c^** |
| Cholesterol,mean(SD),mmol/L | 3.94±1.075 | 4.65±0.811 | 0.201**^c^** |
| Triglyceride,mean(SD),mmol/L | 1.40±0.617 | 1.52±0.419 | 0.465**^a^** |
| High Density Lipoprotein,mean(SD),mmol/L | 1.24±0.357 | 1.21±0.226 | 0.581**^c^** |
| Low Density Lipoprotein,mean(SD),mmol/L | 1.98±0.747 | 2.41±0.698 | 0.357**^a^** |
| DME = Diabetic Macular Edema;RVO-ME = Retinal Vein Occlusion-related Macular Edema; FPG = Fasting Plasma Glucose;SD = Standard Deviation;Independent samples t-tests were used for variables that were normally distributed with homogeneity of variance; otherwise, the Mann–Whitney U test was applied. Chi-square tests were used for categorical variables. *P* < 0.05 is considered statistically significant.  **^a^** Independent samples t-tests; **^b^** x2 test. **^c^** Mann–Whitney U test was applied. | | | |
