# Supplementary material for: Aqueous humour extracellular vesicle membrane protein profiling reveals pathological features of refractory macular edema
Source: Front Cell Dev Biol. 2026 Sep 1;14:1880885. doi: 10.3389/fcell.2026.1880885 (PMC13574810; doi:10.3389/fcell.2026.1880885)
Supplement: Supplementary file 3 [file Supplementaryfile3.docx]

**Table S3. Characteristic of subjects for refractory diabetic macular edema and refractory retinal vein occlusion-related macular edema aqueous humor collection.**

| Group | KEGG pathway | *P.adj* |
| --- | --- | --- |
| Treatment-naïve DME  VS  Refractory DME | Focal adhesion | **< 0.05** |
|  | PI3K-Akt signaling pathway | **< 0.05** |
|  | Rap1 signaling pathway | **< 0.05** |
|  | Ras signaling pathway | **< 0.05** |
|  | Calcium signaling pathway | **< 0.05** |
|  | MAPK signaling pathway | **< 0.05** |
|  | EGFR tyrosine kinase inhibitor resistance | **< 0.01** |
|  |  |  |
| Treatment-naïve RVO-ME  VS  Refractory RVO-ME | Focal adhesion | **< 0.001** |
|  | PI3K-Akt signaling pathway | **< 0.001** |
|  | Rap1 signaling pathway | **< 0.001** |
|  | Ras signaling pathway | **< 0.001** |
|  | Calcium signaling pathway | **< 0.001** |
|  | MAPK signaling pathway | **< 0.001** |
|  | EGFR tyrosine kinase inhibitor resistance | **< 0.01** |
| DME = Diabetic Macular Edema;RVO-ME = Retinal Vein Occlusion-related Macular Edema;AH = Aqueous Humor; *P.adj* < 0.05 is considered statistically significant. | | |
